# Antegrade or retrograde approach for thoracic duct embolization? Inhalation may be part of the answer

**DOI:** 10.1186/s42155-020-00139-w

**Published:** 2020-07-15

**Authors:** Gérald Gahide, Karl Sam

**Affiliations:** 1 Service d’Angioradiologie, Département d’Imagerie Médicale, Centre Hospitalier Universitaire de Sherbrooke, 3001, 12ème avenue Nord, Sherbrooke, Québec J1H 5H3 Canada; 2 Département de Radiologie, Centre Hospitalier Universitaire de Trois Rivières, 1991 Boulevard du Carmel, Trois-Rivières, QC G8Z 3R9 Canada

To the Editor,

We read with great interest the nice case reported by Rott et al. regarding their successful transvenous retrograde thoracic duct embolization (Rott and Boecker 2020). This is a very interesting and promising technique that should probably be considered and used more often. The authors reported some difficulties in navigating the thoracic duct due to its small caliber. We were wondering if they had used any dynamic maneuver to try to open the thoracic duct valves.

A patient was recently referred to our department for the treatment of a high-outpout chylothorax following a right lower lobectomy. After a lengthy and unsuccessful attempt of intranodal lymphangiography, it was decided to try a transvenous retrograde thoracic duct embolization. After localizing the thoracic duct with a left subclavian venography, a retrograde catheterization of the thoracic duct was performed using a 0.021in microcatheter over a glidewire. As Rott et al., we faced several points of resistance when trying to navigate the thoracic duct. Koike et al. suggested that the process of advancing the wire and the catheter in the duct is extensive, especially because of the difficulty in traversing the terminal valves (Koike et al. 2013). We then hypothesized that a negative thoracic pressure induced by inhalation could open the thoracic duct valves as it does with the venous system. Whenever a point of resistance was faced, the patient was asked to take a deep breath. This technique greatly helped the progression of the microcatheter till the cysterna chyli, that was reached in a matter of minutes.

If we were to treat a thoracic duct injury again, it is very likely that we would try a retrograde catheterization first. Though many publications have reported impressive results when performing an intranodal lymphography, our short experience was not that enjoyable. We encountered three main difficulties. First, we didn’t have the chance to use a dedicated syringe to infuse the Lipiodol (Guerbet, France), making the delicate and constant infusion very difficult. Secondly, the 25G needle placed at the corticomedullary interface of the node was very instable, and we lost several beautiful lymphatic channels. Last, we had some lipiodol going directly into the internal iliac venous system due a chylovenous communication that made us stop because of the uncertainty relating to its significance. According to Kariya et al., 76.9% of the patients have a simple cervical thoracic duct, meaning a catheterizable thoracic duct, the remaining of patients having a plexiform anatomy (Kariya et al. 2018). Considering all the hurdles of the lymphography, not to speak of the rate of success for the subsequent percutaneous transabdominal cysterna chyli catheterization, it makes no doubt that it is really worth trying a retrograde approach first. In this regard, we are also interested in knowing if the authors have any recommendation regarding how to localize the thoracic duct.

Sincerely yours,

Gerald Gahide and Karl Sam.

## Data Availability

N/A
